# Origin of the Low Magnetic Moment in Fe_2_AlTi: An Ab Initio Study

**DOI:** 10.3390/ma11091732

**Published:** 2018-09-14

**Authors:** Martin Friák, Anton Slávik, Ivana Miháliková, David Holec, Monika Všianská, Mojmír Šob, Martin Palm, Jörg Neugebauer

**Affiliations:** 1 Institute of Physics of Materials, Academy of Sciences of the Czech Republic, Žižkova 22, CZ-616 62 Brno, Czech Republic; tono.slavik@gmail.com (A.S.); ivanamihalik2@gmail.com (I.M.); Monika.Vsianska@seznam.cz (M.V.); mojmir@ipm.cz (M.Š.); 2 Department of Condensed Matter Physics, Faculty of Science, Masaryk University, Kotlářská 2, CZ-611 37 Brno, Czech Republic; 3 Department of Physical Metallurgy and Materials Testing, Montanuniversität Leoben, Franz-Josef-Strasse 18, A-8700 Leoben, Austria; david.holec@unileoben.ac.at; 4 Central European Institute of Technology, CEITEC MU, Masaryk University, Kamenice 5, CZ-625 00 Brno, Czech Republic; 5 Department of Chemistry, Faculty of Science, Masaryk University, Kotlářská 2, CZ-611 37 Brno, Czech Republic; 6 Max-Planck-Institut für Eisenforschung GmbH, Max-Planck-Str. 1, D-40237 Düsseldorf, Germany; m.palm@mpie.de (M.P.); neugebauer@mpie.de (J.N.)

**Keywords:** Fe_2_AlTi, Fe_2_TiAl, Heusler, magnetism, ab initio, stability, off-stoichiometry, strain

## Abstract

The intermetallic compound Fe${}_{2}$AlTi (alternatively Fe${}_{2}$TiAl) is an important phase in the ternary Fe-Al-Ti phase diagram. Previous theoretical studies showed a large discrepancy of approximately an order of magnitude between the ab initio computed magnetic moments and the experimentally measured ones. To unravel the source of this discrepancy, we analyze how various mechanisms present in realistic materials such as residual strain effects or deviations from stoichiometry affect magnetism. Since in spin-unconstrained calculations the system always evolves to the spin configuration which represents a local or global minimum in the total energy surface, finite temperature spin effects are not well described. We therefore turn the investigation around and use constrained spin calculations, fixing the global magnetic moment. This approach provides direct insight into local and global energy minima (reflecting metastable and stable spin phases) as well as the curvature of the energy surface, which correlates with the magnetic entropy and thus the magnetic configuration space accessible at finite temperatures. Based on this approach, we show that deviations from stoichiometry have a huge impact on the local magnetic moment and can explain the experimentally observed low magnetic moments.

## 1. Introduction

The Fe-Al-Ti ternary system is the basis for materials with a wide range of technologically interesting properties. Notable are, for example, medical applications [1,2], their ability to form oxygen-containing inclusions strenthening steels [3], their potential for high-temperature applications [4,5,6,7,8,9], and more [10,11,12,13,14,15,16]. For a detailed overview, see the review by Palm and Lacaze in Ref. [17]. A subset of these high-temperature materials are two-phase Fe-Al-Ti superalloys (e.g., [18,19,20,21]) containing an off-stoichiometric Fe${}_{2}$AlTi intermetallic compound.

Stoichiometric Fe${}_{2}$AlTi crystallizes in the Heusler L2${}_{1}$-structure. It was included in an extensive theoretical study by Gilleßen and Dronskowski in which they calculated properties of 810 different compounds with either Heusler [22] or inverse Heusler [23] structure type. Elastic properties of Fe${}_{2}$AlTi were recently theoretically studied at ambient conditions [24] as well as under hydrostatic pressures [25]. The thermal expansion coefficient, Debye temperature, and heat capacity at temperatures up to 1200 K and pressures up to 250 GPa were determined [25]. The electronic structure of Fe${}_{2}$AlTi was experimentally analyzed by Kourov et al. [26], and it was found strongly spin-polarized in agreement with theoretical studies (see, e.g., Shreder et al. [27]).

There is a long-lasting discrepancy between the experimental magnetic moment of Fe${}_{2}$AlTi and the theoretical prediction of this quantity, which is nearly an order of magnitude higher. In particular, experimental values of 0.1 $\mu_{B}$ per formula unit (abbreviated as f.u.) reported in Ref. [28] or 0.11 $\mu_{B}$/f.u. in Ref. [29] for *T* = 4.2 K, are significantly lower than the results of density functional theory (DFT) calculations (e.g., 0.9 $\mu_{B}$/f.u. in Refs. [27,30]). This is indeed very interesting because it is well-known that DFT calculations mostly predict the magnetic moment of materials very well. For example, that of Fe is predicted to be 2.25 $\mu_{B}$ [31], matching perfectly the experimental value of 2.22 $\mu_{B}$ [32].

To resolve this issue, we studied in detail how deviations from perfect crystal (e.g., deviations in stoichiometry, internal strain, etc.) affect the local moment. Considering that in typical spin-unconstrained calculations the system always evolves to the spin configuration which represents a local or global minimum at the total energy surface, we turn the investigation around and use constrained spin calculations. Fixing the global magnetic moment to a set of different values, we obtain direct insight into local and global energy minima (reflecting metastable and stable spin phases). Employing these fixed-spin moment (FSM) quantum-mechanical calculations, we determine thermodynamic, electronic, and structural properties of both stoichiometric and off-stoichiometric Fe${}_{2}$AlTi at different magnetic states. Based on these calculations, we explain the discrepancy discussed above.

## 2. Materials and Methods

**Figure 1:**
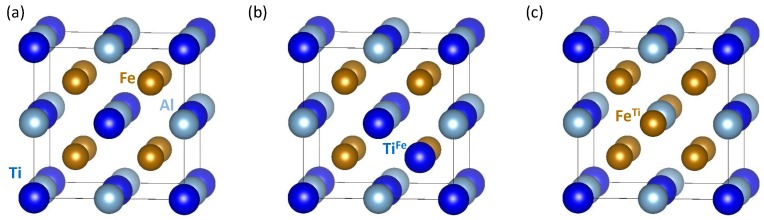
(**a**) Schematic visualization of the cube-shaped 16-atom Fe${}_{2}$AlTi supercell used in our calculations. Two types of substituted states (discussed below) when either (**b**) Ti replaces Fe or (**c**) Fe replaces Ti.

The calculations were performed within the framework of density functional theory [33,34] using the Vienna Ab initio Simulation Package (VASP) [35,36] and projector augmented wave pseudopotentials [37,38]. The exchange and correlation energy was treated in the generalized gradient approximation (GGA) as parametrized by Perdew and Wang [39] using the Vosko–Wilk–Nusair correction [40]. We used a plane-wave energy cut-off of 350 eV with a 10 × 10 × 10 Monkhorst-Pack k-point mesh. The system is described with the help of a cube-shaped 16-atom supercell containing four formula units of Fe${}_{2}$AlTi (see both the stoichiometric case in Figure 1a and supercells with anti-sites in Figure 1b,c).

The FSM approach [31,41,42,43,44,45,46,47] allows the total magnetic moment of a computational supercell to be fixed at a specific value. The local magnetic moments are free to change (given the overall global constraint) regarding their magnitudes as well as their orientations (here only parallel or anti-parallel). When fixing the total magnetic moment of the computational cell, all local magnetic moments are initially oriented in a parallel manner (a ferromagnetic state).

## 3. Results

**Figure 2:**
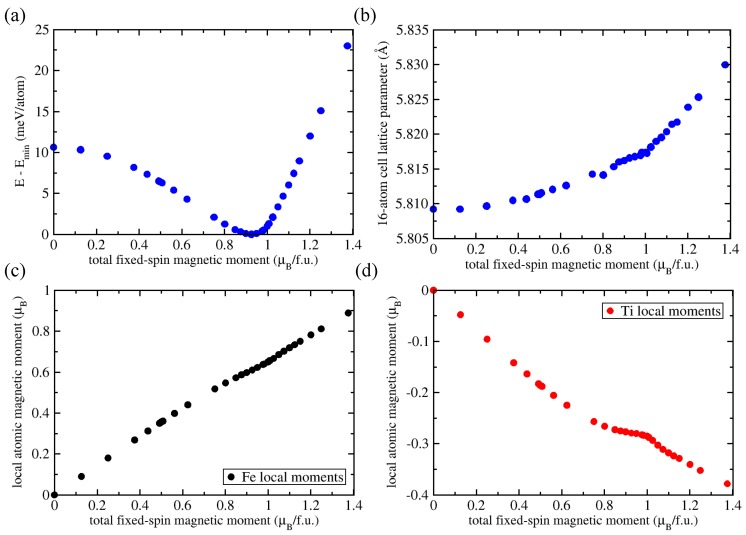
Computed dependencies of (**a**) the total energy (referenced to the lowest obtained energy), (**b**) a 16-atom supercell lattice parameter and local magnetic moments of (**c**) Fe and (**d**) Ti atoms as functions of the fixed-spin value of the total magnetic moment per formula unit.

In order to identify the reason why the DFT computed ground state of Fe${}_{2}$AlTi shows a much higher magnetic moment than is observed experimentally, we systematically searched for additional minima on the total energy landscape. These minima correspond to metastable phases and may correspond to magnetic states that are closer to the experimental one. We therefore calculated the properties of different magnetic states of stoichiometric Fe${}_{2}$AlTi as a function of fixed-spin moment. Figure 2 summarizes the computed energies, lattice parameters, and local magnetic moments of Fe and Ti atoms as functions of the fixed-spin moment. As visualized in Figure 2a, there was only a single minimum. The lowest energy was predicted for a state with the FSM of 0.925 $\mu_{B}$ per formula unit. The local magnetic moments of the Fe and Ti atoms were 0.610 and −0.279 $\mu_{B}$/atom, respectively (the negative sign indicates an anti-parallel orientation—i.e., Fe${}_{2}$AlTi is in a ferrimagnetic state, in contrast to the initial ferromagnetic state of our calculations when all magnetic moments were parallel). The opposite orientation of the local magnetic moment of Ti lowered the energy of the systems and resulted from a full relaxation of all degrees of freedom in our calculations. Our predicted values agreed with those previously reported in Ref. [48] (0.95 $\mu_{B}$/f.u., 0.67 and −0.28 $\mu_{B}$/atom).

Figure 2b shows that the lattice parameter of our cube-shaped 16-atom supercell monotonously increased with increasing fixed-spin moment (except for some small scatter in our data points). The lowest energy state was predicted to have a lattice parameter of 5.817 Å, in agreement with 5.879 Å calculated by Fecher et al. [48]. They also agreed with the experimental values of 5.879 Å reported by Buschow and van Engen [29], as well as 5.858 Å published in Ref. [27] and 5.878 Å found in Ref. [19]. Parts (c) and (d) of Figure 2 depict local magnetic moments of Fe and Ti atoms. The opposite sign of the Fe in Figure 2c and Ti Figure 2d moments implies an anti-parallel orientation. The absolute value of the local magnetic moments of the Fe and Ti atoms increased with increasing FSM value.

Our calculations revealed an interesting feature of the states around the lowest-energy state (see Figure 2d). The local magnetic moments at Ti atoms decreased less steeply just prior to reaching the state with the lowest energy and started to decrease more steeply again only when reaching states with FSM higher than about 1 $\mu_{B}$ per formula unit. In contrast, for these values of the FSM, the local magnetic moments of the Fe atoms showed a significantly smaller deviation from a monotonously increasing trend (see Figure 2c). Aluminium atoms (not shown) were predicted to have negligible local magnetic moments (lower than 0.02 $\mu_{B}$/atom), and were therefore considered in the following discussion as non-magnetic.

We thus conclude that in the case of strain-free stoichiometric Fe${}_{2}$AlTi, only a single energy minimum exists (i.e., metastable configurations with a low spin state do not exist). We now explore alternative mechanisms that may explain the discrepancy between theoretical and experimental data. First, our fixed-spin-moment analysis showed that the non-magnetic state was higher in energy than the lowest-energy magnetic state. However, this difference was only 10.6 meV/atom (see Figure 2a). In fact, its energy was so close to the lowest-energy ferrimagnetic state that it may be thermodynamically stabilized in Fe${}_{2}$AlTi samples already at rather low temperatures. As we have only T = 0 K results, we used Boltzmann statistics to consider approximatively, for example, a temperature of 100 K which was chosen because it is rather close to the magnetic transition temperature of 123 K detected by Buschow and van Engen [29]. The probability of finding the measured samples in a non-magnetic state, instead in the ferrimagnetic lowest-energy one, was significant even at this low temperature (22.6%). Such non-magnetic fluctuations could be easily accommodated by the surrounding ferrimagnetic matrix: the non-magnetic state had a lattice parameter (5.809 Å) very similar to that of the lowest energy ferrimagnetic state (5.817 Å) as seen in Figure 2b. Importantly, while the overall magnetization can be reduced due to statistically quite high probability of non-magnetic states, this probability became significant only at temperatures of at least 30–40 K. On the other hand, the measurements by Buschow and van Engen [29] were performed at 4.2 K. We can therefore also exclude this mechanism as the origin of the low magnetic moment.

**Figure 3:**
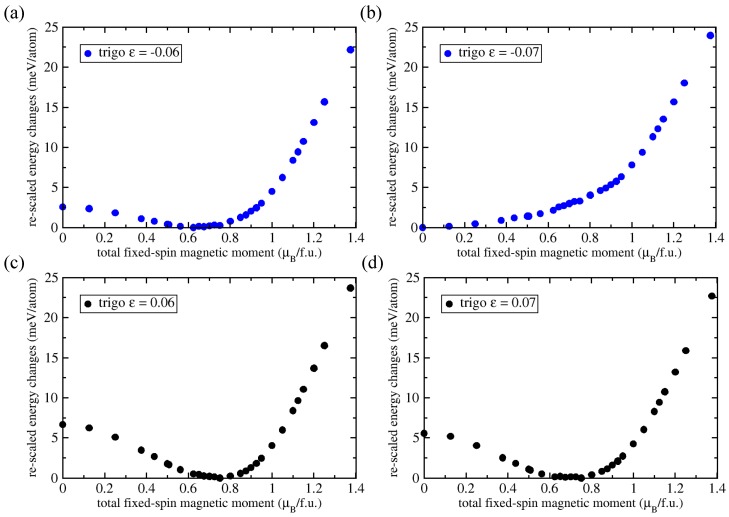
Ab initio calculated energies of trigonally deformed Fe${}_{2}$AlTi for different values of fixed-spin moment (FSM). Results are shown for selected values of strain ε including negative (**a**) −0.06 and (**b**) −0.07 and positive (**c**) 0.06 and (**d**) 0.07. The energies for each value of ε and different values of FSM are shown as differences with respect to the lowest energy for a given value of ε.

Next, we simulated the impact that local strain and deformations may have on the local magnetic moments. We therefore simulated two types of cell-shape deformations. For small strains, these deformations are associated with the cubic-symmetry elastic constants $C_{44}$ and $C^{\prime }$. A trigonal deformation with strain ε modifies the original set of cubic-cell vectors [1, 0, 0], [0, 1, 0], and [0, 0, 1] to a new one [1, ε, ε], [ε, 1, ε] and [ε, ε, 1], tetragonal deformation leads to unit-cell vectors [1–ε/2, 0, 0], [0, 1–ε/2, 0], and [0, 0, 1+ε]. Regarding the terminology, positive strains (e.g., trigonal ones) are tensile along the [111] direction while compressing the crystal within the perpendicular plane. Below, such strains are called tensile. Figure 3 summarizes our results related to trigonally deformed Fe${}_{2}$AlTi for different values of FSM and selected values of strain ε.

As shown in Figure 3a, starting at a strain ε = −0.06 an energy minimum at non-zero FSM was observed. This minimum is related to a ferrimagnetic state. Going to the strain ε = −0.07, the energy minimum corresponds to a non-magnetic state with FSM = 0 (see Figure 3b). This behavior was confirmed for strains ε = −0.075 and −0.080 (not shown). The ferrimagnetic states for positive (tensile) strains (along the [111] direction) had lower magnetic moments compared to the strain-free states (see Figure 3c,d). However, the reduction was much smaller than in the case of negative strains. Fe${}_{2}$AlTi thus exhibited a strong asymmetry between tensile and compressive loading. Importantly, negative strains from −0.07 onwards destabilized the ferrimagnetic state in favor of the non-magnetic one.

It is tempting to assign the zero/vanishingly low magnetic moment to the experimentally observed low moment. Unfortunately, this mechanism became active only at large strains beyond −0.07. Such large compressive strains (along the [111] direction) can only appear locally (e.g., at grain boundaries, close to dislocation cores or other point or extended defects). In a typical experimental sample, the concentration of these extended defects is too low to explain the experimentally observed low magnetic moment. We applied the same approach to tetragonal deformations. However, the local moments were stable for strains up to ±0.080 (not shown). We can therefore also exclude strains as a possible source of low magnetic moments.

**Figure 4:**
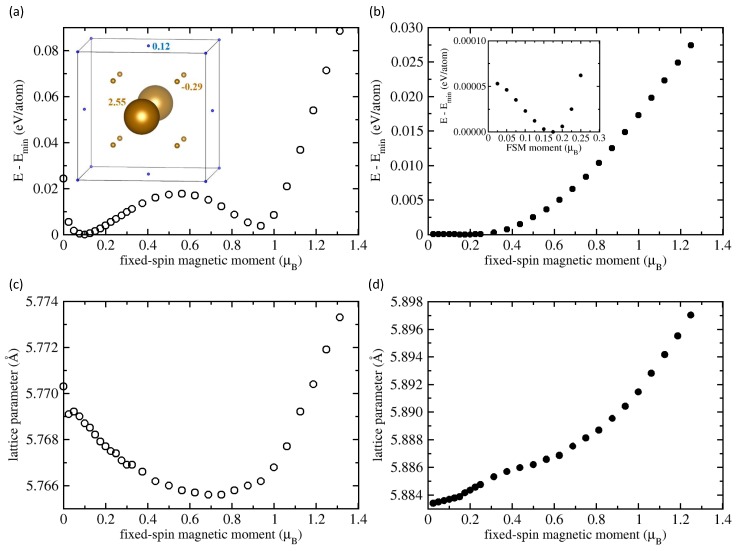
Computed total energies for non-stoichiometric Fe${}_{2}$AlTi. The results for the excess Fe concentration Fe${}_{56.25}$Al${}_{25}$Ti${}_{18.75}$ are shown in (**a**,**c**). For excess Ti, the corresponding results are displayed in (**b**,**d**). The values of the FSM are per four atoms in order to allow for a comparison with the figures visualizing results for the stoichiometric Fe${}_{2}$AlTi discussed above. The inset within part (**a**) shows the local magnetic moments of atoms in the state with the lowest energy (some atoms are shown with their periodic images) and that in part (**b**) magnifies an extremely shallow energy minimum.

As a further mechanism we studied deviations from ideal stoichiometry. Experimentally, it is highly non-trivial to synthesize truly stoichiometric single-phase Fe${}_{2}$AlTi samples. The reason is that during cooling from the melt Laves phases, such as Fe${}_{2}$Ti, form [49,50]. These phases only slowly dissolve when approaching lower temperatures due to sluggish kinetics. Consequently, most Fe${}_{2}$AlTi samples are actually off-stoichiometric. We simulated this situation by introducing off-stoichiometric defects such as Fe${}^{\mathrm{Ti}}$ or Ti${}^{\mathrm{Fe}}$ anti-sites. The calculations were performed with the help of our computational 16-atom supercell. The anti-sites are marked as Ti${}^{\mathrm{Fe}}$ in Figure 1b and Fe${}^{\mathrm{Ti}}$ in Figure 1c, respectively. Our results are depicted in Figure 4. To allow for a direct comparison with the stoichiometric results, the trends are displayed using the same formula unit consisting of four atoms as a reference.

Going to an excess Fe concentration Fe${}_{9}$Al${}_{4}$Ti${}_{3}$ (56.25 at. % Fe and only 18.75 at. % Ti), we found two energy minima (see Figure 4a). The lowest energy was found for a state with fixed-spin moment equal to 0.1 $\mu_{B}$. This state was separated by an energy barrier of 17.7 meV/atom. The higher-lying energy minimum (higher by 3.7 meV/atom) corresponded to a state with the FSM of 0.938 $\mu_{B}$ per four atoms. The presence of two states is related to the presence of two inequivalent types of Fe atoms which exhibit different magnetic states. The off-stoichiometric anti-site Fe atom was in a high-spin state. Its local magnetic moment was 2.55 $\mu_{B}$. Due to the presence of the Fe${}^{\mathrm{Ti}}$ anti-site, the surrounding eight Fe atoms had their local magnetic moment reduced to 0.29 $\mu_{B}$ (see the inset in Figure 4a).

In the lowest energy state, the magnetic moment of the excess Fe atom was anti-parallel to those of regular Fe atoms. The Ti atoms showed a qualitatively similar behavior (their magnetic moments were anti-parallel to those of regular Fe atoms). The lattice parameter decreased for increasing FSM up to the value of FSM = 0.75 $\mu_{B}$ (see Figure 4c). This magneto-volumetric behavior is very different from that of stoichiometric Fe${}_{2}$AlTi (see Figure 2b).

We now study the opposite type of off-stoichiometry (i.e. Ti excess), creating a Ti${}^{\mathrm{Fe}}$ anti-site defect in the 16-atom supercell (see Figure 4b). The single minimum at 0.175 $\mu_{B}$ at the energy surface was extremely shallow—only 0.05 meV/atom lower than the non-magnetic state at zero FSM. Due to the low energy difference, even low temperatures were sufficient to stabilize non-magnetic configurations. Thus, this type of substitution will also lead to states with very low magnetic moments.

## 4. Discussion

The above results clearly indicate that off-stoichiometry is the most likely candidate to resolve the large discrepancy between measured and computed magnetic moments. Inspecting the ternary Fe-Al-Ti phase diagram at temperatures of 800, 900 and 1000 ${}^{\circ }$C (e.g., Ref. [17]), the experimentally determined region of existence of Fe${}_{2}$AlTi does not contain stoichiometric Fe${}_{2}$AlTi. Rather, it contains only Fe-rich (Ti-lean) off-stoichiometric Fe${}_{2}$AlTi phases with a very wide range of excess Fe (even over 25 at. %). Thus, off-stoichiometric regions are very probably present in experimental samples. As discussed in the previous section, off-stoichiometry is realized by anti-site defects. Since these defects are anti-ferromagnetically coupled to the surrounding Fe atoms, such defects reduce the net magnetic moment of the sample. For the concentration of Fe anti-sites considered here (6.25 at. % excess Fe, see Figure 4a), this lowest-energy state has the magnetic moment of 0.1 $\mu_{B}$ per 4 atoms, which is practically identical to the experimental magnetic moments (0.1 $\mu_{B}$ per formula unit reported in Ref. [28] or 0.11 $\mu_{B}$/f.u. in Ref. [29]).

Our conclusion also fits with the interpretation given by Buschow and van Engen [29]. They critically assessed their measured values of the saturation magnetization and the transition temperature. One critical issue they identified was that the value of the magnetic moment in Fe${}_{2}$TiAl is too low for a (relatively high) transition temperature of 123 K. To resolve this issue, they suggested that the Fe${}_{2}$AlTi compound is either Pauli paramagnetic or antiferromagnetic. Our findings, when the anti-site Fe atoms in the Fe-rich off-stoichiometric case have their local magnetic moment anti-parallel to Fe atoms on the regular Fe sublattice, agree with the antiferromagnetic hypothesis of Buschow and van Engen.

## 5. Conclusions

Using DFT fixed-spin-moment calculations we systematically analyzed the thermodynamic stability, structure, and magnetic properties of different magnetic states of stoichiometric and off-stoichiometric Fe${}_{2}$AlTi. Based on these results we could systematically test various mechanisms that could explain the exceptionally low magnetic moment measured for Fe${}_{2}$AlTi. For the stoichiometric case, none of the the mechanisms studied (i.e., trigonal and tetragonal strains, finite-temperature spin excitations) could account for this effect. Considering Fe-rich and Ti-rich off-stoichiometric configurations with an excess of 6.25 at. % of Fe or Ti atoms, the net magnetization drops to very low values. In the case of Fe${}_{2}$AlTi, off-stoichiometric alloys with excess Fe are more relevant because the experimental Fe-Al-Ti phase diagram contains only Fe-rich phases above 800 ${}^{\circ }$C. For the Fe-rich alloy, the energetically preferred state has a net magnetic moment close to the measured one. Our study shows how sensitively is magnetism affected by off-stoichiometries in the alloy composition. Even relatively small changes of a few percent can cause changes of an order of magnitude in magnetic moments. Therefore, a mandatory requirement when comparing ab initio computed moments with experimental values is to critically assess the stoichiometry of the experimental samples.
